# The longitudinal relationship of school climate with adolescent social and emotional health

**DOI:** 10.1186/s12889-021-10245-6

**Published:** 2021-01-23

**Authors:** Mitchell D. Wong, Kulwant K. Dosanjh, Nicholas J. Jackson, Dennis Rünger, Rebecca N. Dudovitz

**Affiliations:** 1 Division of General Internal Medicine and Health Services Research, David Geffen School of Medicine at UCLA, 1100 Glendon Avenue, Suite 850, Los Angeles, CA 90024 USA; 2 Division of General Pediatrics, David Geffen School of Medicine at UCLA, Los Angeles, CA USA

**Keywords:** School climate, Adolescent health, Social-emotional health

## Abstract

**Background:**

Schools and school climate are thought to influence academic outcomes as well as child and adolescent development, health and well-being. We sought to examine the relationship between several aspects of the school climate with adolescent social-emotional health outcomes.

**Methods:**

We analysed data from the Reducing Inequities through Social and Educational change Follow-up (RISE UP) Study, a longitudinal natural experimental study of Los Angeles high school students collected from 2013 to 2018. We analysed data on the portion of the sample that completed the baseline, 10th grade and 11th grade surveys (*n*=1114). Students reported their perceptions of school climate at 10th grade and social-emotional outcomes including grit, self-efficacy, depression, hopelessness, and stress at baseline (9th grade) and at 11th grade. Multivariable regressions adjusted for student and parental demographics and baseline social-emotional states tested associations between school climate and each outcome.

**Results:**

Students who reported being in authoritative school environments in 10th grade, one that is highly supportive and highly structured, had subsequently higher levels of self-efficacy (*p*< 0.001) and grit (*p*=0.01). They also had fewer depressive symptoms (*p*=0.008), and less hopelessness (*p* = 0.01), stress at school (*p*=0.002) and stress about the future (*p*=0.03) reported in 11th grade.

**Conclusions:**

School climate, and particularly an authoritative school environment, is strongly associated with better social-emotional health among adolescents. Relationship with teachers and their disciplinary style may be a focus for future interventions to improve the social-emotional health of children.

## Background

Schools not only influence the academic trajectory of children, but also their social and emotional development and well-being. Indeed, social and emotional school climate is one of the key components of the Centers for Disease Control and Prevention Whole School, Whole Child, Whole Community Model for addressing adolescent and school health [1]. Although many aspects of school climate are associated with positive mental health and wellbeing, the exact mechanisms by which schools influence child and adolescent social and emotional health are not fully understood.

Prior studies investigating the impact of school environments on adolescent social and emotional health have found inconsistent results, in part due to differences in how school climate is measured [2]. Many of these studies have also been limited by several methodologic issues including lack of longitudinal data, the inability to compare multiple aspects of school climate, and limited measures of social and emotional outcomes. Thus, important questions remained unanswered including to what extent school climate is linked to various social-emotional outcomes and which aspects of school climate are the most salient predictors of student’s emotional well-being. Answering these questions is critical to building school environments that facilitate healthy adolescent development.

Hence, we sought to examine several aspects of school climate that might influence student social and emotional health using longitudinal data from a cohort of students followed from beginning of 9th grade through end of 11th grade. In a review of the literature on school climate, Thapa and colleagues identified several dimensions that include safety, relationships with teachers and peers, pedagogical practices, and institutional environment such as the physical surroundings and resources [3]. Of these dimensions, we focused on specific aspects of safety, the disciplinary environment and teacher relationships that we hypothesized might be predictors of social and emotional health outcomes such as self-efficacy, grit, depression, hopelessness and stress. Our focus on the disciplinary environment and teacher and peer relationships was informed by the school social ecology framework, which stresses the importance of inter-personal relationships, the connection that students feel toward the school and the support that students receive in school, all of which promote better academic and health outcomes [4].

## Methods

### Study design and sample

We analysed data from the RISE-UP (Reducing Health Inequalities through Social and Educational Change Follow Up) study, a longitudinal natural experiment designed to recruit similar samples of students who went to high and low-performing schools in order to examine the effects of high-performing schools on health behaviors among low-income, minority adolescents in Los Angeles. We identified five high-performing public charter schools, chosen because admission was determined by random lottery and academic performance at the school-level was in the top tertile of public high schools in Los Angeles County based on 2012 state standardized test scores (Academic Performance Index). The pool of potential eligible subjects were students who applied for 9th grade admission for the fall of 2013 and 2014 to one of the five schools through the admissions lottery and were English or Spanish speaking. Siblings of current students at the school were ineligible since they were granted admission outside the lottery. We then sampled study participants from two groups: those who were accepted for admission (Intervention) and those on the admission wait list (Control). Of the 1509 eligible subjects whom we contacted for the study, 576 Control and 694 Intervention subjects (1270 total) were enrolled and consented to participated in the study (16% refusal rate). Further details of the original study are published elsewhere [5]. The institutional review board at the University of California at Los Angeles approved this study.

### Data collection

Participants completed a baseline, face-to-face, computer-assisted survey from April of 8th grade through October of 9th grade (baseline 9th grade survey). Similar follow-up surveys were conducted at the end of 10th grade and 11th grade. One thousand one hundred fifty-nine students completed the survey in 10th grade and 1114 students completed the survey in 11th grade for an 87.8% retention rate through 11th grade.

At baseline, students reported information on their demographics (gender, race/ethnicity, birthplace, native language, parental characteristics (birthplace, employment and education) and their parent’s parenting style [6, 7]. At baseline and follow-up, students reported depression using the Center for Epidemiologic Studies Depression Scale (CES-D 10) scale [8–10]. Stress at school and about the future was assessed using the Adolescent Stress Questionnaire [11]. We assessed self-efficacy using an 8-item scale, which included items such as “I will be able to successfully overcome many challenges” and “In general, I can obtain outcomes that are important to me” (5 response options ranging from strongly disagree to strongly agree) [12]. We assessed grit using the 8-item scale developed by Duckworth et al., which asks respondents to report whether statements such as “I am a hard worker” and “I often set a goal but then choose to pursue a different one” are like them (5 response options from very much to not at all) [13]. Hopelessness was measured using a 6-item scale, which included items such as “I might as well give up because I can’t make things better for myself” (5 response options from strongly disagree to strongly agree) [14]. We used simple-summated scores for self-efficacy, grit and hopelessness.

In the 10th grade surveys, students were asked about several aspects of school climate. School order refers to the amount of confusion and chaos in the classroom and was assessed using a 6-item scale that we adapted from a measure of chaos in the home environment developed by Matheny and colleagues [15, 16]. A simple summation of the 6-items was used to create a school order measure with higher scores indicating a more orderly and less chaotic school climate. School disciplinary style was adapted from a measure of parenting style and has two component scales, which were support and structure [17]. To aid in the interpretation of the results, we chose to create a measure of school disciplinary style using the same methods as the widely used parenting style [18]. Thus, we divided the support and structure measures into terciles and classified disciplinary style into 5 groups: neglectful (lowest structure tercile and lowest support tercile), indulgent (lowest structure tercile and highest support tercile), authoritarian (highest structure tercile and lowest support tercile), authoritative (highest structure tercile and highest support tercile), and the remainder classified as “average”. We also examined perceptions of school safety, respect for teachers and teacher support for college [19, 20].

### Data analysis

We conducted linear regression analyses to examine the relationship of school climate variables with adolescent social-emotional health outcomes. For these analyses, we pooled the control and intervention groups. Several of the school climate variables (school order, safety, respect for teachers, and teacher support for college) are continuous variables, each with different ranges and standard deviations. To assist in interpreting and comparing the magnitude of the relationships between these predictors and the social-emotional outcomes, we standardized each predictor variable to have a mean of zero and standard deviation of 1. This was achieved for each of the predictors by taking each subject’s predictor variable score, subtracting the sample mean, and then dividing by the standard deviation.

All models were adjusted for student gender (male/female), Latinx ethnicity (Latinx, non-Latinx), US birthplace (US vs. foreign-born), and native English language (yes/no). We also adjusted for parental characteristics—at least 1 parent born in the US (yes/no), employed full time (yes/no), and graduated from high school (yes/no). Parenting style was categorized into 5 groups (neglectful, indulgent, authoritarian, authoritative, and average) using methods described by Steinberg and colleagues [21]. We also adjusted for the relevant baseline social-emotional outcome. For example, we examined predictors of self-efficacy at 11th grade adjusting for self-efficacy reported at 9th grade in the baseline survey. We adjusted for clustering at the 11th grade high school using generalized estimating equations with exchangeable correlation structure. We conducted univariable regression models for each outcome that fully adjusted for all covariates and clustering, but only included one school climate variable at a time. The multivariable regression models included all school climate variables in the same model to test for which measures were most salient to each outcome. STATA 14.0 (College Station, TX) was used for all analyses.

## Results

The RISE-UP sample was comprised of 1270 students at baseline (9th grade) who attended 147 different high schools. Ninety-one percent (1159) completed the first follow-up survey attending 158 high schools at 10th grade, and 88% of baseline sample (1114) complete the second follow up survey and attended 166 high schools at 11th grade. For the portion of the sample that completed the 10th and 11th grade survey (*n*=1114), just under half of the sample were male, 87% were born in the U.S., 90% were Latinx and 40% were native English speakers. Only one-quarter of students had at least one parent born in the U.S., while 88% had at least one parent working full-time, and 51% had a parent who graduated from high school. (Table 1) Compared to the original study sample, the analytic sample was less likely to be male, native English speakers and have at least 1 parent who was born in the U.S. and more likely to be Latinx and have at least 1 parent employed full-time.

**Table 1:** Student demographics, parental characteristics and behavior, school and mental health outcomes

	Analytic Sample	Sample lost to follow up	***P*** value
**N**	1114	156	
**Student demographics**			
Male (%)	46.3	55.1	0.04
Latinx (%)	90.3	82.1	0.002
Born in the U.S. (%)	87.3	90.4	0.27
Native English speaker (%)	39.7	49.4	0.02
**Parental characteristics**			
1 or more parents born in the U.S. (%)	25.1	36.6	0.003
1 or more parents full-time employed (%)	88.0	81.4	0.02
1 or more parents graduated high school (%)			0.25
No	43.5	36.5	
Yes	51.6	58.3	
Unsure	4.8	5.1	
**Parenting style (%)**			0.60
Normal	50.0	49.4	
Authoritative	20.2	25.0	
Authoritarian	9.4	7.7	
Indulgent	8.9	9.0	
Neglectful	11.5	9.0	

Legend: Analytic sample included subjects who completed the 11th grade survey

Table 2 compares students who were offered admission to a high-performing charter school (Intervention) and those who were not (Control) in the random admissions lottery from the original natural experimental design of the RISE-UP Study. Compared to the control group, the intervention group reported more school order (3.38 vs 3.23, *p*< 0.001), more teacher support for college (3.76 vs. 3.64, *p*< 0.001) and more structured school disciplinary style (3.15 vs. 3.09, *p*=0.02). There were no differences between the intervention and control groups in reported school safety, respect for teachers and supportive school disciplinary style. When categorizing school disciplinary into 5 groups (average, authoritative, authoritarian, indulgent and neglectful) based on the strict and supportive disciplinary scales, there was no difference between the intervention and control groups (*p*=0.32).

**Table 2:** Comparison of Intervention and Control students by reported school climate characteristics

	Control	Intervention	***P*** value
**N**	495	619	
**School environment**			
Order (mean)	**3.23**	**3.38**	**< 0.001**
Safety (mean)	2.48	2.51	0.32
**Teacher relationship**			
Respect for teachers (mean)	3.61	3.6	0.70
Teacher support for college (mean)	**3.64**	**3.76**	**< 0.001**
**Structured school disciplinary style** (mean)	**3.09**	**3.15**	**0.02**
**Supportive school disciplinary style** (mean)	0.04	0.12	0.12
**Disciplinary Style Categories** (%)			0.32
Average	50.3	55.1	
Authoritative	17.0	17.0	
Authoritarian	4.0	3.9	
Indulgent	2.8	3.4	
Neglectful	25.9	20.7	

We examined the relationship between school climate factors measured at 10th grade with the social-emotional outcomes at 11th grade. Table 3 shows the results of the univariable regression models in which each school climate variable was analysed in a separate model. These models included the individual-level perceptions of the school climate and adjusted for baseline social-emotional health outcomes at 9th grade and student and parental characteristics. For all of the social-emotional health outcomes, authoritative disciplinary style (high support and high structure) at 10th grade was associated with substantially more favorable outcomes at 11th grade. For example, students who reported an authoritative school climate had more self-efficacy (0.40 sd points, *p*< 0.001), more grit (0.24, *p*=0.002), lower depression (− 0.27, *p*< 0.001), less hopelessness (− 0.26, *p*< 0.001), less stress at school (− 0.30, *p*< 0.001) and less stress about the future (− 0.24, *p*=0.002). Perceptions of school safety was also an important predictor and was associated with better social-emotional health outcomes. For example, a 1 standard deviation increase in the perception of school safety was associated with 0.09 sd increase in self-efficacy (*p*=0.002) and 0.10 sd decrease in depression (*p*< 0.001). Having greater respect for teachers was also associated with higher self-efficacy and grit, and lower hopelessness and stress in school and about the future.

**Table 3:** Univariable models examining the association of individual-level school climate predictors at 10th grade with mental health outcomes at 11th grade

	Self-efficacy (β, 95%CI)	Grit (β, 95%CI)	Depression (β, 95%CI)	Hopelessness (β, 95%CI)	Stress at school (β, 95%CI)	Stress about future (β, 95%CI)
**School environment**						
Order	**0.06 (0, 0.11)**	0.04 (−0.01, 0.10)	**−0.10 (−0.16, − 0.05)**	**−0.05 (− 0.11, 0)**	− 0.04 (− 0.09, 0.02)	− 0.03 (− 0.09, 0.02)
Safety	**0.09 (0.03, 0.14)**	0.04 (− 0.01, 0.10)	**− 0.10 (− 0.15, − 0.04)**	**− 0.09 (− 0.15, − 0.04)**	**−0.10 (− 0.15, − 0.04)**	**−0.12 (− 0.18, − 0.07)**
**Teacher relationship**						
Respect for teachers	**0.12 (0.06, 0.18)**	**0.07 (0.02, 0.13)**	−0.05 (− 0.11, 0)	**− 0.10 (− 0.16, − 0.05)**	**−0.07 (− 0.13, − 0.02)**	**−0.09 (− 0.14, − 0.03)**
Teacher support for college	**0.08 (0.02, 0.14)**	0.02 (− 0.04, 0.07)	**− 0.06 (− 0.12, − 0.01)**	**− 0.08 (− 0.13, − 0.02)**	− 0.04 (− 0.1, 0.02)	−0.04 (− 0.09, 0.02)
**Disciplinary Style**						
Average	0	0	0	0	0	0
Authoritative	**0.40 (0.25, 0.56)**	**0.24 (0.09, 0.40)**	**−0.27 (− 0.42, − 0.12)**	**−0.26 (− 0.42, − 0.11)**	**−0.30 (− 0.46, − 0.15)**	**−0.24 (− 0.39, − 0.09)**
Authoritarian	−0.16 (− 0.45, 0.13)	− 0.10 (− 0.37, 0.18)	−0.09 (− 0.37, 0.20)	0.05 (− 0.23, 0.33)	0.01 (− 0.27, 0.30)	−0.26 (− 0.54, 0.03)
Indulgent	0.11 (− 0.20, 0.43)	− 0.10 (− 0.41, 0.21)	0.15 (− 0.17, 0.46)	− 0.06 (− 0.38, 0.25)	0.04 (− 0.28, 0.35)	0 (− 0.32, 0.31)
Neglectful	−0.11 (− 0.25, 0.02)	−0.06 (− 0.19, 0.08)	0.11 (− 0.03, 0.24)	0.05 (− 0.08, 0.19)	− 0.05 (− 0.19, 0.08)	−0.04 (− 0.18, 0.09)

Univariable models include each school climate variable individually in separate models, adjusted for student demographics and parental characteristics as well as social-emotional characteristics at 9th grade (baseline)

The outcomes and the predictors school order, safety, respect for teachers, teacher support for college are continuous variables that have been standardized to mean of 0 and sd=1

Results in bold indicate *p*< 0.05

We examined the school climate variables together in one multivariable model to examine which variables had the strongest association with social-emotional health outcomes. As shown in Table 4, individual-level perceptions of the school disciplinary style remained the strongest predictor of social-emotional health outcomes, controlling for other school climate variables as well as baseline social-emotional health and student and parent characteristics. Authoritative school disciplinary style (high support and high structure) was associated with more self-efficacy and grit and less depression, hopelessness and stress. Authoritarian disciplinary style (low support and high structure) was also associated with lower stress about the future (− 0.31 sd points, *p*=0.03). School safety was associated lower stress at school (0.08, *p*=0.006) and about the future (− 0.11, *p*< 0.001), while more school order was associated with less depression (− 0.06, *p*=0.03).

**Table 4:** Multivariable models examining the association of individual-level school climate predictors at 10th grade with mental health outcomes at 11th grade

	Self-efficacy (β, 95%CI)	Grit (β, 95%CI)	Depression (β, 95%CI)	Hopelessness (β, 95%CI)	Stress at school (β, 95%CI)	Stress about future (β, 95%CI)
**School environment**						
Order	− 0.02 (− 0.08, 0.04)	0.01 (− 0.05, 0.06)	**−0.06 (− 0.12, 0)**	0 (− 0.06, 0.05)	0.01 (− 0.05, 0.07)	0.01 (− 0.05, 0.07)
Safety	0.04 (− 0.02, 0.10)	0.01 (− 0.05, 0.07)	**−0.06 (− 0.12, 0)**	−0.05 (− 0.11, 0)	**−0.08 (− 0.14, − 0.02)**	**−0.11 (− 0.17, − 0.05)**
**Teacher relationship**						
Respect for teachers	**0.06 (0, 0.12)**	0.05 (−0.01, 0.11)	0.02 (− 0.05, 0.08)	−0.06 (− 0.12, 0.01)	−0.04 (− 0.11, 0.02)	**−0.06 (− 0.13, 0)**
Teacher support for college	0.03 (− 0.04, 0.09)	−0.02 (− 0.08, 0.04)	−0.03 (− 0.09, 0.03)	−0.03 (− 0.10, 0.03)	0 (− 0.07, 0.06)	0.01 (− 0.05, 0.07)
**Disciplinary Style**						
Average	reference	reference	reference	reference	reference	reference
Authoritative	**0.37 (0.21, 0.53)**	**0.21 (0.05, 0.38)**	**−0.21 (−0.37, − 0.06)**	**−0.21 (− 0.37, − 0.05)**	**−0.26 (− 0.42, − 0.10)**	**−0.18 (− 0.34, − 0.02)**
Authoritarian	−0.14 (− 0.43, 0.14)	−0.1 (− 0.38, 0.18)	−0.13 (− 0.41, 0.16)	0.02 (− 0.27, 0.30)	−0.03 (− 0.31, 0.26)	**−0.31 (− 0.6, − 0.03)**
Indulgent	0.11 (− 0.21, 0.42)	−0.09 (− 0.40, 0.23)	0.14 (− 0.17, 0.45)	−0.06 (− 0.38, 0.26)	0.04 (− 0.28, 0.35)	0 (− 0.32, 0.31)
Neglectful	−0.05 (− 0.19, 0.10)	−0.02 (− 0.17, 0.12)	0.05 (− 0.10, 0.19)	−0.03 (− 0.18, 0.11)	−0.12 (− 0.27, 0.02)	−0.13 (− 0.28, 0.01)

Each multivariable model includes all school climate variables in one model, adjusted for student demographics and parental characteristics as well as social-emotional characteristics at 9th grade (baseline)

The outcomes and the predictors school order, safety, respect for teachers, teacher support for college are continuous variables that have been standardized to mean of 0 and sd=1

Results in bold indicate *p*< 0.05

We conducted a sensitivity analysis examining depression as a dichotomous variable using the CES-D 10 cutoff ≥10, and 21% of the sample met this cutoff at 11th grade. The results were similar compared to the analysis of depression as a continuous variable (results not shown).

## Discussion

The present study provides strong evidence that students’ perception of several aspects of the school climate are associated with adolescent social-emotional health over time. In contrast, prior studies have not consistently shown a relationship between school climate and mental health measures [2]. Prior studies have also been more limited in scope looking at global measures of school climate and only a few mental health outcomes such as depression, suicidal ideation and anxiety. Many studies have relied on cross-sectional analyses, thus making it difficult to rule out the possibility of reverse causality. Although the present study is observational in nature, we were able to examine the longitudinal relationship between several measures of school climate with several different measures of social and emotional health. By adjusting for baseline social-emotional outcomes, our analyses also decrease the possibility of reverse causality, i.e. that social-emotional factors are influencing perceptions of the school environment. Having surveyed students in over 150 different high schools, our results reflect the experience of students in a wide range of environments. Consequently, our findings extend our understanding about the relationship between students’ perception of the school climate and adolescent social-emotional health and suggest a close connection exists.

We found that teacher relationships and disciplinary style may be particularly important protective factors contributing to adolescent well-being. Similar to the parenting literature, perceiving an authoritative school environment, one that is highly supportive and highly structured, is associated with better outcomes. In addition, attending schools in which students have more respect for teachers and interact with teachers who provide strong support for college attendance is also linked to better social-emotional outcomes. These findings are important because they may indicate specific aspects of school climate that might be targeted by future interventions to support adolescent social-emotional health. Many school-based social and emotional programs focus on individual students, attempting to strengthen students’ coping strategies, impulse control, mindfulness, empathy and communication skills [22–24]. While these individual-level interventions are important, fewer interventions focus on school-level factors that might improve adolescent outcomes, such as factors that support teacher-student relationships and authoritative disciplinary style. Indeed, the association between authoritative disciplinary style and better social-emotional outcomes suggests that our current knowledge about effective parenting interventions might be applicable to teachers and other school-related adults to improve behavior management and enhance their relationship with students.

Ideally, schools not only prepare our children to succeed academically, but also prepare them to be resilient, well-adjusted individuals. Prior studies have largely focused on adverse mental health outcomes such as depression and stress [2]. Thus, the present study also contributes to the literature by examining positive social-emotional health outcomes, such as grit and self-efficacy. Prior studies about these social-emotional traits like grit, also known as “soft skills,” suggest that their presence strongly predicts better life outcomes including higher rates of employment, greater income, lower rates of divorce, and better medication adherence [25–28].

The World Health Organization has recommended schools adopt a more holistic approach to child development and well-being [29]. Our findings support the idea that improving school climate might be a strategy to improve student grit, self-efficacy and other aspects of student well-being. Keshavarz and colleagues have suggested a number of strategies that schools might take to shape their school climate and improve student well-being such as creating more structure and having clear rules, promoting positive relationships with peers and teachers, and creating feedback loops that incorporate health outcomes into the design of school policy and function [30]. Some of these approaches have been implemented in some interventions with some positive effects [31, 32].

Our study has several limitations. First, our study was observational in design, thus it is not possible to determine if the relationship between climate and social-emotional outcomes is causal. Second, the majority of our sample were from Latino, immigrant families living in under-resourced neighborhoods of Los Angeles. Thus, the sample and the schools that our participants attended may not generalize to other populations. Of the 1509 students whom we attempted to recruit for the original study, 239 (16%) refused participation. While this refusal rate is low, we do not know if this biased our sample in any way. For the present study, we analyzed 88% (1114/1270) of the sample that was retained in the study through the end of 11th grade, and those who were lost to follow-up were more likely to be male and have parents who were not fully employed, perhaps representing a group at higher risk for worse social-emotional outcomes. We analysed individual perceptions of school climate rather than school-wide characteristics, and thus, it is possible that depression and other social-emotional states could influence perceptions of school climate. Despite this limitation, our longitudinal data allowed us to control for baseline social-emotional states thus reducing the possibility of reverse causality.

## Conclusion

We found strong evidence that students’ perceptions of the school climate factors are associated with adolescent social-emotional outcomes, which supports the idea that schools are more than a means to transmit knowledge or cognitive skills. Schools are social environments that facilitate important relationships with peers and adults. Similar to relationships with parents and family, these school relationships may have important effects on student well-being and resiliency. Hence we should be attuned to the “social education” students receive at school and construct school environments that facilitate adolescents’ social-emotional health.

## Data Availability

The datasets generated and/or analysed during the current study are not publicly available because they contain sensitive information and potential subject identifiers but are available from the corresponding author on reasonable request.

## Abbreviations

RISE UP: Reducing Inequities through Social and Educational change Follow-up
CES-D: Center for Epidemiologic Studies Depression Scale

## References

[1] Lewallen TC, Hunt H, Potts-Datema W, Zaza S, Giles W (2015). The whole school, whole community, whole child model: a new approach for improving educational attainment and healthy development for students. J Sch Health.

[2] Kidger J, Araya R, Donovan J, Gunnell D (2012). The effect of the school environment on the emotional health of adolescents: a systematic review. Pediatrics..

[3] Thapa A, Cohen J, Guffey S, Higgins-DAllesandro A (2013). A review of school climate research. Rev Educ Res.

[4] Waters SK, Cross DS, Runions K (2009). Social and ecological structures supporting adolescent connectedness to school: a theoretical model. J Sch Health.

[5] Dudovitz RN, Chung PJ, Reber S, Kennedy D, Tucker JS, Shoptaw S (2018). Assessment of exposure to high-performing schools and risk of adolescent substance use: a natural experiment. JAMA Pediatr.

[6] Baumrind D (1966). Effects of authoritative parental control on child behavior. Child Dev.

[7] Lamborn SD, Mounts NS, Steinberg L, Dornbusch SM (1991). Patterns of competence and adjustment among adolescents from authoritative, authoritarian, indulgent, and neglectful families. Child Dev.

[8] Radloff LS (1977). The CES-D scale: a self-report depression scale for research in the general population. Appl Psychol Meas.

[9] Björgvinsson T, Kertz SJ, Bigda-Peyton JS, McCoy KL, Aderka IM (2013). Psychometric properties of the CES-D-10 in a psychiatric sample. Assessment..

[10] Sieving RE, Beuhring T, Resnick MD, Bearinger LH, Shew M, Ireland M (2001). Development of adolescent self-report measures from the National Longitudinal Study of adolescent health. J Adolesc Health.

[11] Byrne DG, Davenport SC, Mazanov J (2007). Profiles of adolescent stress: the development of the adolescent stress questionnaire (ASQ). J Adolesc.

[12] Chen G, Gully SM (2001). Validation of a new general self-efficacy scale. Organ Res Methods.

[13] Duckworth AL, Quinn PD (2009). Development and validation of the short grit scale (grit-s). J Pers Assess.

[14] Bolland JM (2003). Hopelessness and risk behaviour among adolescents living in high-poverty inner-city neighbourhoods. J Adolesc.

[15] Wong MD, Chung PJ, Hays Ron D, Kennedy DP, Tucker JS, Dudovitz RN (2019). The social economics of adolescent behavior and measuring the behavioral culture of schools. J Child Fam Stud.

[16] Matheny AP, Wachs TD, Ludwig JL, Phillips K (1995). Bringing order out of Chaos: psychometric characteristics of the confusion, hubbub, and order scale. J Appl Dev Psychol.

[17] Lau C, Wong M, Dudovitz R (2018). School disciplinary style and adolescent health. J Adolesc Health.

[18] Steinberg L, Lamborn SD, Darling N, Mounts NS, Dornbusch SM (1994). Over-time changes in adjustment and competence among adolescents from authoritative, authoritarian, indulgent, and neglectful families. Child Dev.

[19] Steinberg MP, Allensworth E, Johnson DW (2011). Student and Teacher Safety in Chicago Public Schools.

[20] Luppescu S, Hart H, Rosenkranz T, Montgomery N, Sporte S, Sebring PB (2007). Consortium on Chicago School Research 2007 Survey Reports for Chicago Public Schools.

[21] Steinberg L, Lamborn SD, Dornbusch SM, Darling N (1992). Impact of parenting practices on adolescent achievement: authoritative parenting, school involvement, and encouragement to succeed. Child Dev.

[22] Blewitt C, Fuller-Tyszkiewicz M, Nolan A, Bergmeier H, Vicary D, Huang T (2018). Social and emotional learning associated with universal curriculum-based interventions in early childhood education and care centers: a systematic review and meta-analysis. JAMA Netw Open.

[23] Blewitt C, O’Connor A, Morris H, Mousa A, Bergmeier H, Nolan A (2020). Do curriculum-based social and emotional learning programs in early childhood education and care strengthen teacher outcomes? A Systematic Literature Review. Int J Environ Res Public Health.

[24] Šouláková B, Kasal A, Butzer B, Winkler P (2019). Meta-review on the effectiveness of classroom-based psychological interventions aimed at improving student mental health and well-being, and preventing mental illness. J Prim Prev.

[25] Heckman JJ, Stixrud J, Urzua S (2006). The effects of cognitive and noncognitive abilities on labor market outcomes and social behavior. J Labor Econ.

[26] Heckman JJ, Rubinstein Y (2001). The importance of noncognitive skills: lessons from the GED testing program. Am Econ Rev.

[27] Eskreis-Winkler L, Shulman EP, Beal SA, Duckworth AL (2014). The grit effect: predicting retention in the military, the workplace, school and marriage. Front Psychol.

[28] Peña PA, Pérez-Díaz I, Pulido-Ayala AK, Osorio-Landa HK, López-Navarro JM, Duckworth AL (2019). Association of Grit Scores with Treatment Adherence and Biomarkers in patients with type 2 diabetes. JAMA Netw Open.

[29] World Health Organization WHO (1996). School health promotion: Development of health promoting schools: A framework for action.

[30] Keshavarz N, Nutbeam D, Rowling L, Khavarpour F (2010). Schools as social complex adaptive systems: a new way to understand the challenges of introducing the health promoting schools concept. Soc Sci Med.

[31] Bonell C, Allen E, Warren E, McGowan J, Bevilacqua L, Jamal F (2018). Effects of the learning together intervention on bullying and aggression in English secondary schools (INCLUSIVE): a cluster randomised controlled trial. Lancet..

[32] Shinde S, Weiss HA, Varghese B, Khandeparkar P, Pereira B, Sharma A (2018). Promoting school climate and health outcomes with the SEHER multi-component secondary school intervention in Bihar, India: a cluster-randomised controlled trial. Lancet..

